# Optimized Workflow for On-Line Derivatization for Targeted Metabolomics Approach by Gas Chromatography-Mass Spectrometry

**DOI:** 10.3390/metabo11120888

**Published:** 2021-12-18

**Authors:** Raphaela Fritsche-Guenther, Yoann Gloaguen, Anna Bauer, Tobias Opialla, Stefan Kempa, Christina A. Fleming, Henry Paul Redmond, Jennifer A. Kirwan

**Affiliations:** 1 Berlin Institute of Health (BIH) @ Charité—Universitätsmedizin Berlin, BIH Metabolomics Platform, 13125 Berlin, Germany; Raphaela.fritsche@charite.de (R.F.-G.); yoann.gloaguen@mdc-berlin.de (Y.G.); anabauer@ual.com (A.B.); tobias.opialla@mdc-berlin.de (T.O.); 2 Core Unit Bioinformatics, Berlin Institute of Health (BIH) @ Charité— Universitätsmedizin Berlin, 10117 Berlin, Germany; 3 Max Delbrück Center for Molecular Medicine (MDC) in the Helmholtz Association, Berlin Institute of Medical Systems Biology, 10115 Berlin, Germany; Stefan.kempa@mdc-berlin.de; 4 Department of Academic Surgery, Cork University Hospital, T12 DFK4 Cork, Ireland; christinafleming49@gmail.com (C.A.F.); henry.redmond@hse.ie (H.P.R.)

**Keywords:** automated derivatization, on-line derivatization, optimization, gas-chromatography mass spectrometry, validation, metabolomics, quality assurance (QA), quality control (QC)

## Abstract

Using manual derivatization in gas chromatography-mass spectrometry samples have varying equilibration times before analysis which increases technical variability and limits the number of potential samples analyzed. By contrast, automated derivatization methods can derivatize and inject each sample in an identical manner. We present a fully automated (on-line) derivatization method used for targeted analysis of different matrices. We describe method optimization and compare results from using off-line and on-line derivatization protocols, including the robustness and reproducibility of the methods. Our final parameters for the derivatization process were 20 µL of methoxyamine (MeOx) in pyridine for 60 min at 30 °C followed by 80 µL *N*-Methyl-*N*-trimethylsilyltrifluoracetamide (MSTFA) for 30 min at 30 °C combined with 4 h of equilibration time. The repeatability test in plasma and liver revealed a median relative standard deviation (RSD) of 16% and 10%, respectively. Serum samples showed a consistent intra-batch median RSD of 20% with an inter-batch variability of 27% across three batches. The direct comparison of on-line versus off-line demonstrated that on-line was fit for purpose and improves repeatability with a measured median RSD of 11% compared to 17% using the same method off-line. In summary, we recommend that optimized on-line methods may improve results for metabolomics and should be used where available.

## 1. Introduction

Gas chromatography-mass spectrometry (GC-MS) has been widely used for the analysis of highly polar compounds such as amino acids, sugars, sugar alcohols and phosphorylated compounds. Derivatization of the sample is normally necessary to convert the metabolites into their volatile counterparts to facilitate analysis by GC-MS. Derivatization reduces polarity, increases thermal stability and improves peak shape, resolution and intensity [1]. Many derivatization methods are available while methoximation (MeOx) followed by silylation is one of the most widely used methods [2]. The MeOx reaction converts free carbonyl functional moieties (C=O) into oxime derivatives (CH_2_ON) and prevents formation of cyclic structures especially from carbohydrates and steroids. This reduces the number of possible stereoisomers per compound and increases sensitivity of detection. Pyridine is used as both a solvent and as a catalyst in this reaction. Silylation is then typically performed to make compounds more volatile. The most common silylation reagent is MSTFA (*N*-Methyl-*N*-trimethylsilyltrifluoracetamide) which substitutes active hydrogens on the metabolite with a trimethyl-silyl group [-Si(CH_3_)3-]. A further advantage of silylation is the ease-of-use and that the derivatization reaction can be performed at low temperatures.

Most of the protocols to measure polar metabolites are designed for manual (hereafter off-line) derivatization. Major challenges with off-line derivatization are that it is time consuming, and most derivatization products are somewhat unstable. Since most off-line derivatization is performed batch wise, it also leads to variable equilibration times since the samples are waiting different lengths of time in the autosampler before analysis. Thus, derivatization of more than a small batch of samples at once can result in a high variability among the samples [2]. Multiple peaks are usually produced when silylation is incomplete. Factors like temperature, time of derivatization and the volume of the derivatization agent contribute to the reaction speed and completeness of derivatization. Derivatization conditions therefore directly impact on the repeatability and reproducibility of sample analyzes. 

An automated (hereafter on-line) derivatization method can be beneficial. It allows a consistent derivatization time of samples prior to analysis, potentially giving more robust and reproducible data [3]. The on-line workflow also allows the largely unattended analyzes of large sample series. The results are independent of operator and location and should result in highly reproducible data sets.

*Zarate et al*. (2016) [1] have previously presented an automated derivatization protocol by GC-MS using the commercially available software Maestro from Gerstel GmbH. The performance of on-line and off-line derivatization was tested in wine and plasma. However, no parameter optimization regarding chemical volumes, incubation time and temperature or equilibration time was performed. *Abbiss et al.* (2015) [2] have also investigated automated derivatization of both silylating agent and batch versus just-in-time derivatization but did not optimize setting parameters [4]. The aim of the current study was to develop a fully automated derivatization method which could then be applied for targeted analysis of common human and animal matrices. We describe the method optimization steps and compare the data obtained from both off-line and on-line derivatization methods. The developed automated derivatization method should perform at least as well as the off-line derivatization in its detection and reproducibility capability.

## 2. Results

All the samples from off-line and on-line derivatization were run in scan mode to obtain both targeted and untargeted metabolic profiles. However, in the current study we selected 42 specific metabolites from the central carbon metabolism (CCM) to cover the biological classes glycolysis, amino acids and metabolites from the tricarboxylic cycle (TCA) as well as some chemical classes herein named as other (nucleobases/nucleosides, phosphate compounds, short chain fatty acids, sugar alcohols, carboxylic acids and glycerol metabolites) that we have previously targeted in other studies (Supplementary Material Table S1) [5,6,7]. The detection of these compounds after preparation with off-line and on-line derivatization protocols were directly compared.

In metabolomics, data reproducibility is usually reported as relative standard deviation (RSD in %) of a repeated sample measurement, e.g., a quality control (QC) sample analyzed multiple times over several batches. A commonly accepted maximum tolerance for GC-MS in metabolomics studies is 30% [8,9]. An RSD of less than 15% is considered as very good reproducibility [10].

**Figure 1:**
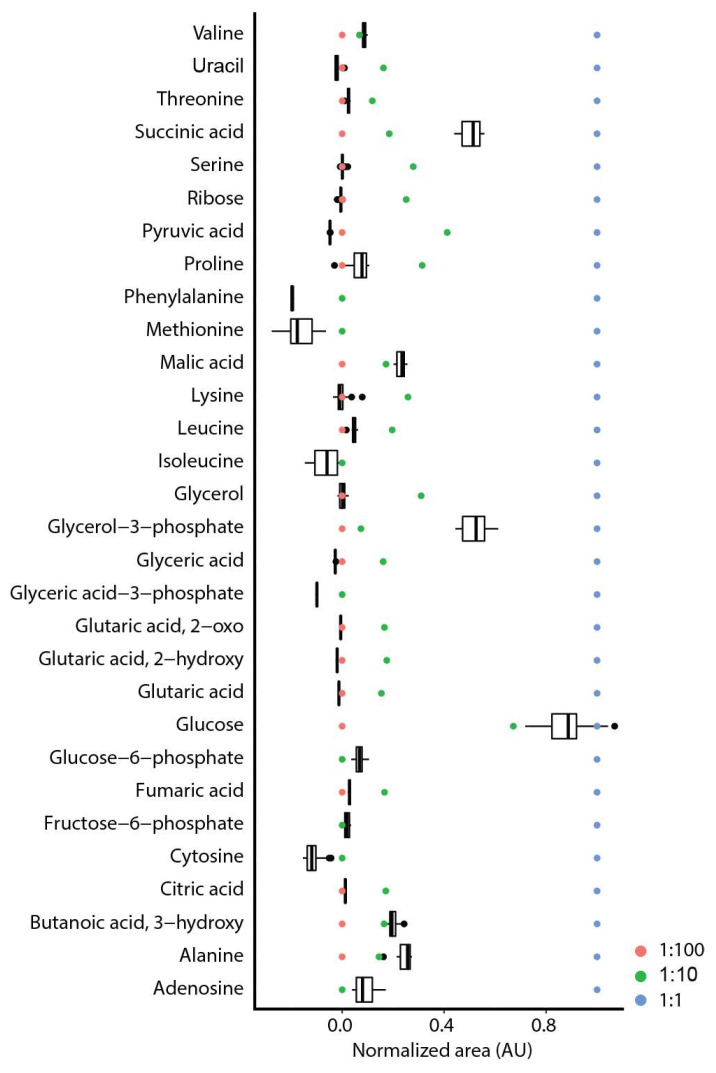
Concentration range of 30/42 detected compounds in mouse liver. A calibration mixture at high (1:1), middle (1:10) and low (1:100) concentration was used for comparison (one for calibration mixture and *n* = 18 for liver). A minimum/maximum normalization was performed on the calibration mixture for the single compounds.

For our optimization studies, a calibration mixture was used containing the metabolites of interest in designated low (1:100 diluted), middle (1:10 diluted) and high concentration (1:1 not diluted) solutions (Supplementary Material Table S2). For the parameter optimization we focused on the 1:10 calibration mixture. When using plasma or serum as matrix, most of the detected metabolites were detected in the low concentration range of our calibration mixture (low signal intensity) (Supplementary Material Figures S1 and S2), whereas for liver, the compounds were more in a medium range (Figure 1). Only glucose was detected at high intensities in mouse liver samples.

### 2.1. Optimization of the Derivatization Parameters

**Figure 2:**
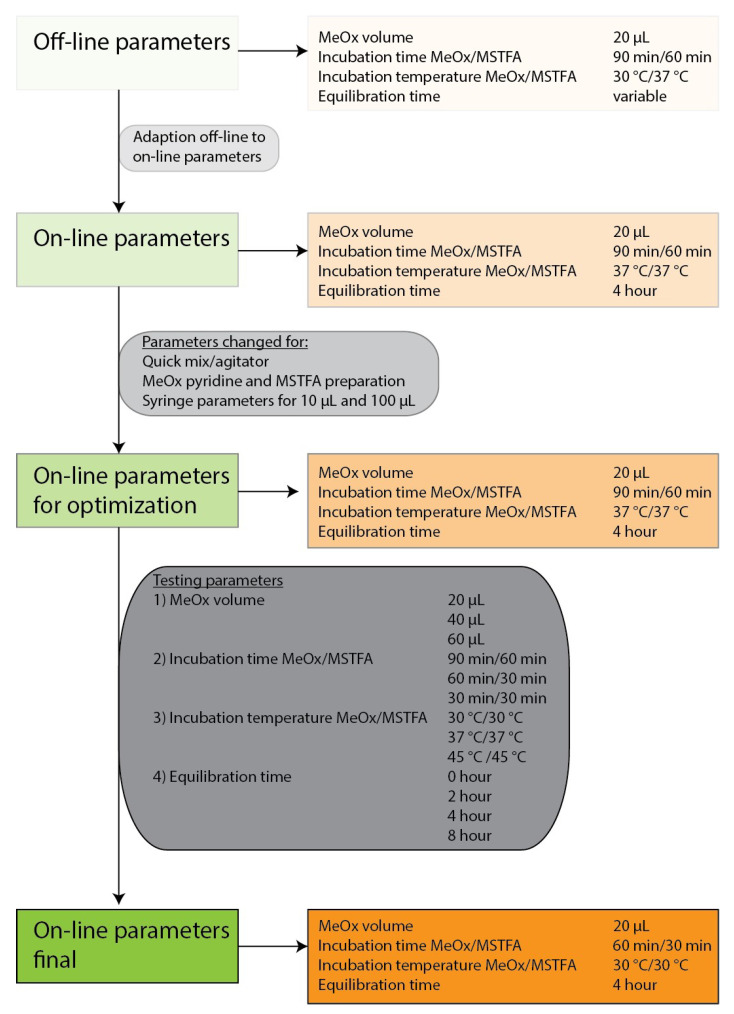
Workflow for off-line and on-line parameter optimization.

We started with our current off-line protocol (Supplementary Material Table S3). A few modifications were immediately required to enable it to work as an on-line method due to technical limitations of the multipurpose sampler (MPS). This included changing the incubation temperature for both the MeOx/pyridine and MSTFA incubation derivatization step from 30 °C to 37 °C. Changing the temperature of the agitator between the two steps is difficult and time consuming during a sequence, so both reactions were conducted under the same temperature conditions. An equilibration time of 4 h was set for the on-line method, while it continued to be variable for samples prepared batch-wise in the off-line. In addition, we optimized some other parameters to improve the derivatization results (Supplementary Material Table S3, see on-line optimized). This includes the preparation of MeOx/pyridine and MSTFA aliquots at the beginning of the sequence for all samples, the removal of the quick mix shaking step after addition of MeOx/pyridine or MSTFA and the reduction of the agitator speed from 750 rpm to 250 rpm. Syringe parameters were changed to reduce the number of misinjections and to avoid air bubbles during aspiration of the liquids (Supplementary Material Figure S3). The workflow of the parameter optimization for MeOx/pyridine volume, incubation time and temperature and the equilibration time of the sample is shown in Figure 2.

#### 2.1.1. MeOx Volume

First, we tested three different MeOx volumes (20 µL, 40 µL, 60 µL all at 40 mg MeOx per mL pyridine concentration). An alkane mixture for reliable retention index calculation was included. Alkane 32 was used to analyze the instrumental drift throughout the run. Cinnamic acid was used as an internal standard for compound normalization. An overall reduction of around 30% in the average peak area was observed for alkane 32 and cinnamic acid as the volume of MeOx increased (mean area alkane 32 for 20 µL = 3.0 × 10^5^, 40 µL = 2.4 × 10^5^, 60 µL = 2.1 × 10^5^; mean area cinnamic acid for 20 µL= 6.5 × 10^4^, 40 µL = 5.3 × 10^4^, 60 µL = 4.6 × 10^4^). This was likely a dilution effect due to increasing total volume. A measure of 20 µL MeOx provided the overall best results for the 1:10 calibration mix (Table 1). The median RSD was 17% for 20 µL MeOx volume compared to 27% and 33% for 40 µL and 60 µL (*p* = 8.53 × 10^−7^ by Kruskal-Wallis (KW) test), respectively. In line, the median RSD per biological class was lower when using 20 µL MeOx compared to 40 µL and 60 µL. It was found that RSDs were significantly worse when 60 µL was used compared to both 20 µL and 40 µL (*p* = 1.23 × 10^−6^ by KW test). The numbers of compounds detected were 33 using 20 µL, 33 using 40 µL and 32 when using 60 µL MeOx. Adenine and glucose-6-phosphate were only detected in the 20 µL MeOx condition, while fructose-6-phosphate was only detected using 40 µL MeOx. Cytosine could be detected in the 40 µL and 60 µL MeOx condition but not for 20 µL. The measurement response, as measured by the normalized peak area for each compound, was, on average, greater per compound when using 20 µL of MeOx compared to 40 µL or 60 µL (Supplementary Material Figure S4). For the 1:1 calibration mix dilution, a lower median RSD was found for 60 µL MeOx (21% for 20 µL, 17% for 40 µL, 15% for 60 µL), albeit with the loss of up to two compounds in the 60 µL condition (36 compounds) compared to both 20 µL MeOx (37 compounds, adenine could be detected) and 40 µL MeOx (38 compounds, adenine and adenosine could be detected) (Supplementary Material Table S4). For the 1:100 calibration mix dilution, the overall best conditions were observed using 60 µL of MeOx. However, two compounds more (phenylalanine, 4-amino butanoic acid) could be detected in the 20 µL and 40 µL conditions (24, 24, 22 for 20 µL, 40 µL, 60 µL, respectively). The median RSD was much higher in 20 µL and 40 µL compared to 60 µL (32%, 34% and 20% for 20 µL, 40 µL and 60 µL, respectively) which may be due to signal interference from other substances in the same mixture (so-called “matrix effects”).

#### 2.1.2. Incubation Time

In a second step, the different incubation times for both MeOx/pyridine and MSTFA were tested. Our first observation was that results were highly dependent on individual metabolites (although there were trends within a class) and on the overall metabolite concentrations. Our original off-line method used an incubation time of 90 min for MeOx, then 60 min for MSTFA (90/60 min), but for the on-line method we found the best condition for the 1:10 calibration mix dilution was 60/30 min (Table 1). Repeatability, as measured by RSD, is better overall (median RSD 15%), and better for virtually all of the individual biological classes when a 60/30 min incubation protocol is used (Supplementary Material Table S5). This combination also resulted in the highest number of detected compounds (34). Tyrosine, methionine and fructose-6-phosphate could be detected only when using these conditions, although lysine and ribose-5-phosphate were only detected using the 30/30 min protocol and citric acid was only detected using the 90/60 min protocol. Violin plots of the ratio of normalized peak areas of the detected compounds visualize how areas (and therefore sensitivity) changes overall between two different methods. The ratios of normalized peak areas were similarly distributed when using the indicated incubation times, however, with slight exceptions (Supplementary Material Figure S4). Concentration affected both reproducibility, detection and the optimal derivatization times. For the 1:1 calibration mix, the repeatability was marginally better when 90/60 min was used (12% compared to 13% and 18% for 30/30 min and 60/30 min, respectively), while the number of detected compounds was marginally higher when the 30/30 min protocol was used (37, 34, 36 for 30/30 min, 60/30 min and 90/60 min, respectively). Overall, and perhaps unsurprisingly, this more concentrated mixture performed best for both repeatability and detection. For the 1:100 calibration mix dilution, compound RSDs were shown with a 16% decrease in the median RSD for the 90/60 min incubation condition, alongside a corresponding 20% decrease in the number of detected compounds (24, 25 and 19 compounds detected for 30/30 min, 60/30 min and 90/60 min, respectively).

#### 2.1.3. Incubation Temperature

As a third optimization parameter, we analyzed different incubation temperatures for both MeOx/pyridine and MSTFA. For the on-line method it is not possible to change the temperatures in the agitator within a few minutes. Therefore, one optimal temperature for both derivatization steps were analyzed. Once again, we saw an effect of metabolite concentration on the results. The optimized temperature for the 1:10 calibration mix dilution was 30 °C (Table 1). The median RSD was 10% (10% 37 °C, 21% 45 °C; 30 °C versus 45 °C *p* = 1.37 × 10^−2^ and 37 °C versus 45 °C *p* = 7.88 × 10^−6^) and 38 compounds could be detected (37 at 37 °C, 36 at 45 °C). Notably, derivatization of amino acids and glycolytic phosphates as well as the two carboxylic acids lactic acid and pyruvic acid performed significantly better when 37 °C was used. The analysis of the peak area revealed a high spread in the 30 °C compared to 37 °C with half of the compounds showing a decrease and half an increase in the peak area (Supplementary Material Figure S4). For 1:1 calibration mix dilution the lowest median RSD (27% 30 °C, 16 % 37 °C, 16 % 45 °C) and 3 extra compounds were found using 37 °C (Supplementary Material Table S6). The number of detected compounds were broadly similar for 1:1 and 1:10 calibration mixes (range 36–39), while a notable decrease was seen in the 1:100 quant across all incubation temperatures (range 29–32) reflecting the substantially lower concentration. This is also the likely explanation for the observed higher median RSDs seen in this group.

#### 2.1.4. Equilibration Time

Four different equilibration times were tested (0 h, 2 h, 4 h and 8 h). The lowest median RSD was found for 0 h (11%), while 4 h and 8 h both had median RSDs of 15% (Table 1). The highest median RSD was found when using 2 h of equilibration time (21%). This is also reflected in the median RSDs per biological group. Significant differences were found comparing 0 h to 2 h (*p* = 3.10 × 10^−5^), 2 h to 4 h (*p* = 4.49 × 10^−3^) and 2 h to 8 h (*p* = 3.43 × 10^−2^). The number of compounds detected was equal for 2 h and 4 h (36) and 0 h and 8 h (34). The 0 h of equilibration time showed slightly lower peak areas compared to 4 h, while for 2 h the areas were equivalent to 4 h. The measurement from the 8 h equilibration time showed a wider spread but with a trend of lower peak areas compared to 4 h (Supplementary Material Figure S4). For the 1:1 calibration mix dilution, 2 and 8 h showed the lowest median RSD (both 11%; 21% 0 h and 28% 4 h) (Supplementary Material Table S7). A measure of 38 compounds were detected with an equilibration time of 4 h and 36 for the other tested conditions. For the 1:100 calibration mix dilution the best condition is 8 h of equilibration time giving a median RSD of 16% and 27 detected compounds (median RSD 29%, 21%, 35% and 25, 24 and 27 compounds for 0 h, 2 h and 4 h, respectively). To reduce the total run time, we compromised on using 4 h for equilibration of the samples after the derivatization process. This enables detection of two additional compounds but sacrifices some technical reproducibility. This may need to be changed if matrices with predominantly low or high compound concentrations are used. 

To see which tested condition had the major effects the absolute RSD change and the percent of increase per tested condition were analyzed. Our optimization method suggested that the determination of the MeOx volume makes the largest overall difference to reproducibility (a 16% absolute difference in RSD and a 95% increase in area) although the incubation temperature had a 12% absolute difference in median RSD between the highest (22%) and lowest (10%) median RSD values measured.

In summary, our final defined parameters for the 1:10 calibration mix dilution was 20 µL of MeOx/pyridine for 60 min at 30 °C followed by 80 µL MSTFA for 30 min at 30 °C combined with 4 h of equilibration time.

### 2.2. Repeatability and Reproducibility

Like any analytical field, metabolomics requires methods that are robust and reproducible. To test the repeatability of this method, 45 replicates of human plasma and 18 replicates of mouse liver were analyzed using the final parameters (Table 2, Supplementary Material Tables S8 and S9). To test the reproducibility of the method, 9 quality control samples (serum samples pooled after extraction from healthy and sick patients) were analyzed over 3 batches within the scope of a biological study, i.e., realistic conditions (Table 2 and Supplementary Material Table S10). Number of metabolites detected, number of missing values and median RSD were used as indicators of sensitivity, method robustness (including derivatization completeness) and reproducibility.

The repeatability test in plasma showed a median RSD of 16% ranging from 11% (valine) to 28% (alanine) for individual compounds for 44/45 samples. One sample was lost due to a misinjection. Ten metabolites showed a median RSD of less than 15% indicating a very good repeatability. For the individual biological classes, a median RSD of 18% was found for amino acids, 13% for glycolysis and 15% for TCA cycle. The analysis of the MeOx and trimethylsilyl (TMS) groups revealed an RSD of 15% for metabolites with 4 TMS groups (3 metabolites, all RSD 15%), 20% for metabolites with 3 TMS groups (8 metabolites, RSD range 13–52%), 25% for metabolites with 2 TMS groups (12 metabolites, RSD range 12–50%) and 37% for metabolites with 1 TMS group (4 metabolites, RSD range 19–37%). MeOx products showed a median RSD of 16% (2 metabolites, RSD 12% and 20%). On average 23 compounds were found in the 44 plasma samples. Of the data, 1% were missing values. 

The repeatability test in liver showed a median RSD of 10% ranging from 2% (malic acid and succinic acid) to 56% (4-amino butanoic acid) for individual compounds. A total of 22 metabolites showed a median RSD of < 15% indicating a very good repeatability. For the individual biological classes, a median RSD of 13% was found for amino acids, 21% for glycolysis and 3% for TCA cycle. The analysis of the MeOx and TMS groups revealed an RSD of 7% for metabolites with 4 TMS groups (6 metabolites), 21% for metabolites with 3 TMS groups (16 metabolites), 22% for metabolites with 2 TMS groups (17 metabolites) and 13% for metabolites with 1 TMS group (3 metabolites). MeOx products showed a median RSD of 22% over 7 metabolites). On average, 37 compounds were found in the 18 liver samples. No missing values were present.

For the reproducibility study, pooled serum samples were used. The overall sequence length was spread over three batches and comprised 115, 115 and 118 injections in total for batch 1, 2 and 3, respectively, including washes (*n* = 26, *n* = 25, *n* = 29), equilibration samples (*n* = 2 per batch), identification mixtures (*n* = 4), quantification mixtures (*n* = 8), long term internal reference QC liver samples (*n* = 3), pooled QC samples (*n* = 10) and human serum samples (*n* = 59, *n* = 60, *n* = 62). The biological results from this study are detailed in another paper. Two QCs were measured at the beginning and the end of each batch. The rest of the QCs were equally distributed over the whole sequence. The reproducibility test of the pooled serum samples showed a consistent intra-batch median RSD of around 20% ± 1% while the inter-batch variability across three batches was 27%. For individual metabolites, the measured variability was considerably higher: intra-batch range 3% to 69%, inter-batch range 15% to 77%. Equivalent numbers of compounds could be detected in all three batches.

### 2.3. Comparison of on-Line to off-Line Derivatization

**Figure 3:**
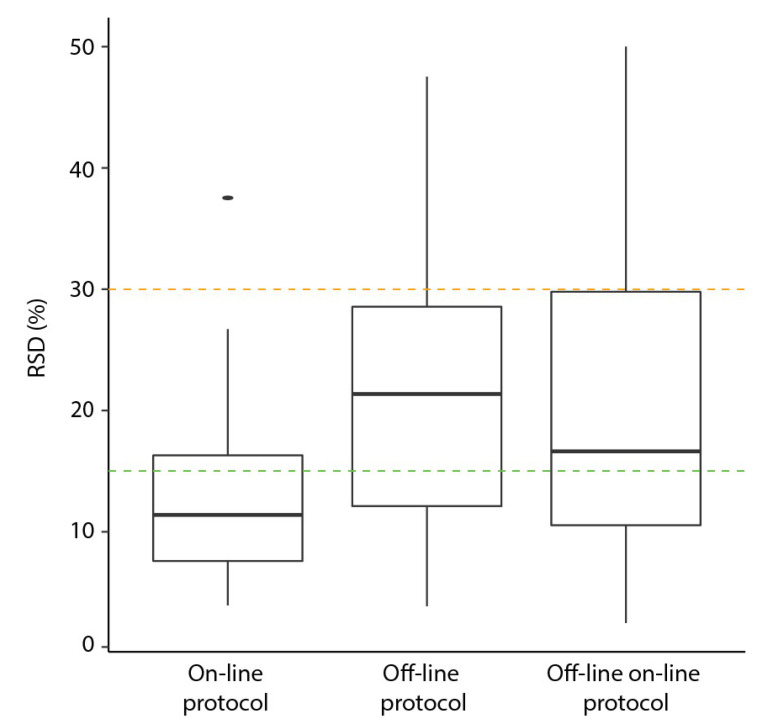
Distribution of individual metabolite relative standard deviation (RSD in %) for the different protocols used. The orange and green lines indicate RSD thresholds of 30% and 15%, respectively.

To benchmark the results, we compared on-line to off-line derivatization (Table 3). For the off-line experiments, both the original and the newly adapted for on-line derivatization protocols (including new time and temperature parameters) were used. Human plasma samples were aliquoted, extracted, derivatized and analyzed for each condition (each *n* = 9). The mean number of detected compounds were similar across all three protocols (23.4, 24.2 and 24.4 for on-line, off-line (original) and off-line with on-line parameters (OLOLP), respectively); however, the number of missing values was slightly but not significantly higher when using the on-line protocol (6% compared to 3% for off-line and OLOLP). A typical chromatogram of a plasma sample prepared with on-line method is shown in Supplementary Material Figure S5. For the compounds common to all methods, the lowest median RSD was found in the on-line derivatization (11%) compared to off-line (21%, *p* = 4.51 × 10^−4^) and OLOLP (17%, *p* = 4.23 × 10^−2^). The lowest individual compound RSD (2% for phenylalanine) was found for the OLOLP protocol (compared to 4% for both pyruvic acid and phenylalanine for on-line and off-line, respectively). By contrast, proline had the highest individual compound RSD (38%) in the on-line method. The highest individual RSD for off-line protocols was found for ribose (48%) and adenine (50%) for OLOLP. The distribution of the individual RSDs is shown in Figure 3. This demonstrates that there are compound-specific differences in performance of protocols. When analyzing the different methoximation and silylation products, lower median RSDs were found in the on-line protocol for 1 TMS (20%, 21%, 22%), 2 TMS (16%, 23%, 21%), 3 TMS (9%, 22%, 16%), 4 TMS (7%, 13%, 11%) and MeOx groups (20%, 29%, 45%) compared to original and OLOLP, respectively. Four of our measured metabolites were detected with two different TMS groups (leucine, phenylalanine, threonine and valine). The ratio between the two TMS groups detected was higher using the on-line compared to both off-line conditions (Supplementary Material Table S11). When all metabolites were accounted for, a significantly higher normalized peak area was found for on-line compared to off-line (*p* = 1.50 × 10^−2^) and on-line compared to OLOLP (*p* = 2.53 × 10^−2^) conditions.

In summary, the optimized parameters indicate that the on-line derivatization method was fit for purpose and may have some advantages over off-line methods in repeatability and reproducibility.

## 3. Discussion

The aim of the current study was to develop a fully automated derivatization method for targeted metabolic profiling of different matrices which allows consistent derivatization time of samples prior to injection thus giving more robust and reproducible data. More traditional off-line derivatization necessitates variable within-batch equilibration times after derivatization, which affects the reproducibility and quality of the acquired data [3]. Equilibration time refers to the time following the derivatization steps where the sample is left to stabilize at room temperature before injection. Equivalent equilibration times for all samples is the major advantage of on-line derivatization and should lead to more consistent results and potentially better sensitivity, compared to off-line derivatization. Equilibration times allow more complete derivatization of compounds with slower reaction times but may also result in the degradation of some less stable derivatized compounds. Optimal equilibration times are therefore compound dependent. An on-line method allows better control of both the derivatization process and time before injection of the samples thus reducing technical variation between the samples. 

We optimized our derivatization protocol using MeOx/pyridine and MSTFA as derivatizing agents. Since the derivatization time was longer than the GC-MS analysis time, the use of a commercial software (Maestro software available for Gerstel) further enabled us to minimize total instrument time by allowing us to overlap the derviatization sequences of individual samples and still consistently inject samples at the same stage of derivatization. Methoximation is performed to inhibit ring formation of reducing sugars by protection of the aldehyde and ketone groups [11]. It stabilizes ring sugars in an “open” linear formation and results in two stereoisomers (cis and trans), which will later be separated by GC-MS. A l derivatization time ensures the completion of methoxymation; however, this could also result in progressive degradation of heat labile metabolites [12]. In addition, we observed that the overall areas decrease by around 30% with increasing volumes of MeOx. We attribute this to a dilution effect due to the increased volume of solvent in the final sample. Our optimization method suggested that the determination of the MeOx volume and incubation temperature makes the largest overall difference to reproducibility. With our optimized derivatization protocol, we anticipate the method could be used for the analysis of a wide range of biological samples with different matrices. 

Our presented optimized on-line method showed a good repeatability for different compound classes including amino acids, sugars and organic acids. An inter-batch study was conducted comparing pooled quality control samples over three batches. The reproducibility over a long sequence (more than 100 injections at once) was in an acceptable range (27%). This is particularly impressive when considering that the matrix itself (human serum) can be challenging to analyze due to a large dynamic range and many very low intensity peaks of interest. Plasma and serum have proven to be particularly challenging matrices to analyze, with the analysis dominated with large MSTFA and contaminant peaks, while peaks of interest are often at low intensities. In some cases, compounds were detectable, but had poor peak shape. Poor peak shape makes quantifying peak area potentially unreliable, and these peaks are best excluded for further statistical analysis. Since poor peak shapes tend to be reflected in poorer RSDs, these peaks are subsequently excluded as part of the normal quality control RSD filtering which we routinely use in our lab for biological datasets. However, it is still judicious to validate important biological findings by re-examining raw data. 

A further advantage of on-line derivatization is that a greater number of samples can be analyzed in one batch. Minor disadvantages of on-line derivatization are the higher costs (larger volume of chemicals, glass vials and magnetic lids are required), more training required for sequence programming and the potential loss of some specific metabolites. However, these disadvantages are relatively small compared to the overall advantages offered by the convenience and reproducibility of the method.

We were surprised that the concentration of a particular mixture should affect the optimal derivatization parameters. While for some factors, this may be availability of derivatizing agent or dilution, other factors such as temperature are less easily explainable, although there may also be a concentration dependent general evaporation or vaporization effect of specific metabolites that occurs at higher temperatures or over longer times.

We had also anticipated a greater range of results between the metabolic classes, especially where there were different numbers of maximum TMS groups. We did indeed see some differences in optimal derivatization conditions between the classes, and we also saw that the median RSDs were variable between the TMS groups. However, the number of TMS groups did not seem to affect the measured optimal parameters overall, probably because there were only four compounds where there was consistently more than one TMS state.

Our developed and optimized on-line derivatization method allows the immediate derivatization of samples prior to injection giving more robust and reproducible data [3]. Additionally, the method saves personnel time with both sample preparation and later data processing. Further development of the instrument method may improve results for particular matrices, e.g., serum.

## 4. Materials and Methods

All chemicals used were analytical grade.

### 4.1. Extraction of Calibration Standards

A mix of commercially available, biologically relevant calibration standards (calibration mix dilution) were used in our method development tests [13]. The mix was diluted to make high (1:1, undiluted), middle (1:10) and low (1:100) metabolite concentration mixes. A biphasic extraction of the standards was performed as follows: 1 mL of methanol (MeOH, Chemsolute/Th.Geyer, Berlin, Germany, cat-number 1428.1000 LC-MS grade), chloroform (CHCl_3_, Sigma-Aldrich, St. Louis, MO, USA, cat-number 132950 >99.8% grade) and water (H_2_O, VWR, Radnor, Pennsylvania, US, cat-number 83645.290) in a 5:2:1 ratio plus 2 µg/mL cinnamic acid (Sigma-Aldrich, St. Louis, MO, USA, cat-number C80857) was added to the calibration standards and shaken for 15 min at 4 °C. A measure of 0.5 mL of H_2_O was added to introduce phase separation followed by shaking for 15 min at 4 °C and centrifugation at 4.149× *g* for 10 min at 4 °C. Next, 500 µL aliquots of the polar phase was collected in GC-MS champagne vials and dried over night at 30 °C with 1.550× *g* at 0.1 mbar vacuum using a rotational vacuum concentrator (RVC 2-33 CDplus, Christ, Osterode am Harz, Germany). The dried aliquots were stored at −80 °C until use when they were prepared as described below.

### 4.2. Plasma and Serum Extraction

Human plasma was drawn from healthy volunteers in accordance with the ethical standards of the institutional review board of the Charité-Universitätsmedizin Berlin (ethical approval EA2/046/17). Human serum was obtained from healthy patients and patients with colorectal cancer. Ethical approval was prospectively approved by CREC (Clinical Research Ethics Committee of the Cork Teaching Hospitals), Cork, Ireland [reference number: ECM5(12)-11/09/07& ECM3-05/06/18]. Frozen plasma or serum was thawed, vortexed and 25 µL of the plasma or serum was added into prechilled and aliquoted MeOH (112.5 µL) followed by 15 s of vortexing. Afterwards, a MeOH/CHCl_3_ mixture (987.5 µL) including 6 µg/mL cinnamic acid and 382.5 µL H_2_O was added, vortexed for 15 s and left on ice for 10 min to separate the biphasic mixture. Samples were centrifuged at 2.560× *g* for 20 min at 4 °C and then left at room temperature for 10 min to equilibrate. Next, 300 µL of the polar phase were collected in GC-MS champagne vials and dried over night at 30 °C at 1.550× *g* at 0.1 mbar vacuum using a rotational vacuum concentrator (RVC 2-33 CDplus, Christ, Osterode am Harz, Germany). Pooled quality control samples from all analyzed serum samples were generated after extraction and used in this study to analyze reproducibility.

### 4.3. Liver Extraction

Mouse liver (obtained from already sacrificed mice at animal facility at Max Delbrück Center, Berlin, Germany) was weighed and homogenized using the Precellys 24 tissue lyzer (Bertin Instruments, Montigny-le-Bretonneux, France). A measure of 1 mL of MeOH:CHCl_3_:H_2_O in a ratio of 5:2:1 including 2 µg/mL cinnamic acid was added to 50 mg of tissue and incubated for 15 min at 4 °C with continuous shaking. Afterwards, 0.5 mL of H_2_O was added and incubated for 15 min at 4 °C with continuous shaking following centrifugation at 4.149× *g* for 5 min at 4 °C. Next, 500 µL aliquots of the polar phase were collected in GC-MS champagne vials and dried over night at 30 °C with 1.550× *g* at 0.1 mbar vacuum using a rotational vacuum concentrator (RVC 2-33 CDplus, Christ, Osterode am Harz, Germany). The dried aliquots were stored at −80 °C until use when they were prepared as described below.

### 4.4. GC-MS Metabolomics Measurement of Key Central Carbon Pathway Metabolites

Extracts were removed from the freezer and dried in a rotational vacuum concentrator (RVC 2-33 CDplus, Christ, Osterode am Harz, Germany) for 60 min before further processing to ensure there was no residual water which may influence derivatization efficiency.

#### 4.4.1. On-line Derivatization

A Gerstel autosampler (Multi Purpose Sampler; Mülheim, Germany) and the commercially available software Maestro (version 1.4.40.0) was used for automatically overlapping derivatization and GC-MS acquisition. Derivatization parameters tested included (1) MeOx (cat-number 226904) in pyridine (cat-number 270970) volume (both Sigma-Aldrich, St. Louis, MI, USA), (2) incubation time for MeOx in pyridine and MSTFA (Macherey-Nagel; Düren, Geramany, cat-number 701270.201), (3) incubation temperature for MeOx in pyridine and MSTFA and (4) equilibration time of the sample.

**Figure 4:**
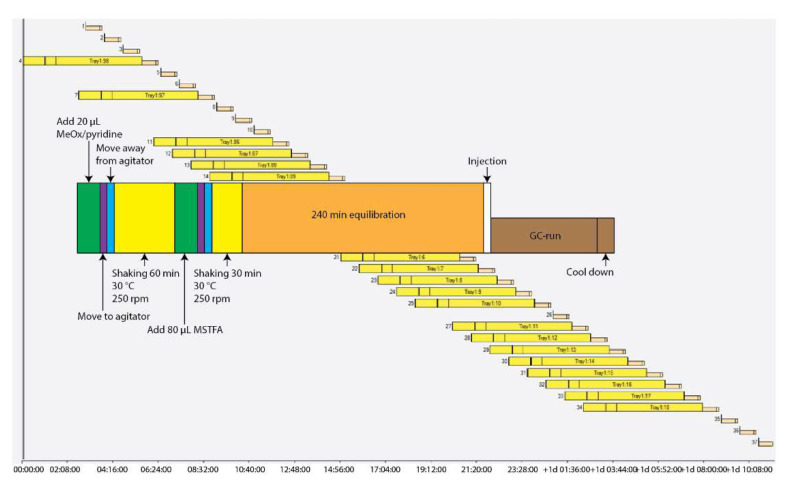
Schematic of a typical automatic derivatization sequence. The multicolored diagram in the foreground displays the order of events for a single typical sample. The background diagram shows how these events are overlapped with different samples to maximize GC-MS instrument time. The GC-MS runs that are not attached to a derivatization sequence (salmon colored) represent MSTFA washes which are injected throughout the run to assess for any carryover.

A sequence was generated to set up an automated methoxymation and trimethylsilyl derivatization run (Figure 4). Champagne vials (Zinsser Analytic, Eschborn, Germany) with magnetic lids (Fisher Scientific, Hampton, VA, USA) were used. Dried extracts were dissolved in 20 µL (40 µL or 60 µL for optimization studies) of MeOx solution (40 mg/mL in pyridine) and incubated for indicated time, temperature and agitator shaking, followed by the addition of 80 µL of MSTFA containing an alkane mix (20 µL per 1 mL of MSTFA, C10 (cat-number 457116), C12 (cat-number 297879), C15 (cat-number P3406), C17 (cat-number 51578), C19 (cat-number 747158), C22 (cat-number 134457, C28 (cat-number O504), C32 (cat-number 44253) and C36 (cat-number H12572) in 50% MeOH, all Sigma-Aldrich, St. Louis, MO, USA) and incubated for indicated time, temperature and agitator shaking. After finishing the derivatization, the vial remained on tray for indicated times (equilibration) before contents were injected for analysis. 

#### 4.4.2. Off-line Derivatization

Two off-line derivatization methods were used to compare to the on-line derivatization method. One used the same protocol as used for the on-line derivatization, except it was done batch-wise by hand (and so equilibration times varied across the batch). The other followed our original protocol and was also performed batch-wise [5]. For off-line derivatization dried extracts were dissolved in 20 µL of MeOx solution (40 mg/mL in pyridine) and incubated for 90 min and 30 °C in a thermoshaker (800 rpm), followed by the addition of 80 µL of MSTFA (which contained the same alkane mix as for on-line) and incubated for 60 min at 30 °C in a thermoshaker (800 rpm). For the off-line with on-line parameters (OLOLP), dried extracts were dissolved in 20 µL of MeOx solution (40 mg/mL in pyridine) and incubated for 60 min at 30 °C in a thermoshaker (300 rpm), followed by the addition of 80 µL of MSTFA plus alkane mix and incubation for 30 min at 30 °C in a thermoshaker (300 rpm). After centrifugation for 10 min at maximum speed, 30 µL was transferred to GC-MS champagne vials and closed with appropriate lids (Labconco, Kansas City, MO, USA).

#### 4.4.3. Instrumentation

An identification mixture for reliable compound identification was prepared and derivatized in the same way, and an alkane mixture for reliable retention index calculation was included [14]. Metabolite analysis was performed on a Pegasus HT GC-TOFMS System (LECO Corporation, St. Joseph, MN, USA) complemented with an auto-sampler (Gerstel MPS DualHead with CAS4 injector, Mühlheim an der Ruhr, Germany). The samples were injected in split mode (split 1:5, injection volume 1 µL) in a temperature-controlled injector with a baffled glass liner (Gerstel, Mühlheim an der Ruhr, Germany). The following temperature program was applied during sample injection: for 2 min the column was allowed to equilibrate at 68 °C, then the temperature was increased by 5 °C/min until 120 °C, then by 7 °C/min up to 200 °C, then by 12 °C/min up to a maximum temperature of 320 °C which was then held for 7.5 min. Gas chromatographic separation was performed on an Agilent 7890 (Agilent Technologies, Santa Clara, CA, USA), equipped with a VF-5 ms column (Agilent Technologies, Santa Clara, CA, USA) of 30 m length, 250 µm inner diameter, and 0.25 µm film thickness. Helium was used as the carrier gas with a flow rate of 1.2 mL/min [13]. The spectra were recorded in a mass range of 60 to 600 m/z with 10 spectra/sec. The GC-MS chromatograms were processed with ChromaTOF software (LECO Corporation, St. Joseph, MN, USA) including baseline assessment, peak picking and computation of the area. A minimum signal to noise ratio of 10 was used to pick peaks. Data were initially automatically annotated and integrated using ChromaTOF software Version 4.72.0.0, but then manually checked and adjusted to assume correct peak annotations and integrations.

#### 4.4.4. Data Analysis

The data were exported and merged by an in-house written R-script. A total of 42 targeted metabolites from the central carbon metabolism covering glycolysis, tricarboxylic cycle, amino acids, nucleobases/nucleosides, pentose phosphate pathway, short chain fatty acids, sugar alcohols, carboxylic acids and glycerol pathway were selected for the reference search (Supplementary Material Table S1) [13]. Microsoft Excel (Microsoft Excel 2013 15.0.5327.1000) and R Studio (version 1.3.1056) were used for data analysis and Adobe Illustrator (version 16.0.0) was used for visualization. For the calibration mix, dilutions 1:1, 1:10 and 1:100 were each measured in a total of 5 replicates. The metabolites were considered valid when they appeared in minimum of *n* = 3 replicates per condition. The peak area of each metabolite was calculated by normalization to the internal standard cinnamic acid. The measured derivatives were summarized. Evaluation of optimal parameters was achieved by the number of compounds, missing values, individual, median RSDs and median RSDs per biological class. For reproducibility studies using pooled serum samples the overall sequence length was 115, 115, 118 injections containing washes (*n* = 26, *n* = 25, *n* = 29), equilibration samples (*n* = 2), identification mixtures (*n* = 4), quantification mixtures (*n* = 8), liver QC samples (*n* = 3), pooled QC samples (*n* = 10) and human serum samples (*n* = 59, *n* = 60, n = 62), for batch 1, 2 and 3, respectively. Two QCs were measured at the beginning and the end of each batch. The rest of the QCs was equally distributed over the whole sequence. Number of metabolites detected, number of missing values and median RSD were used as indicators of sensitivity, method robustness (including derivatization completeness) and reproducibility, respectively. A Kruskall-Wallis test followed by a Tukey Kramer multiple comparison test was conducted to assess the significance of differences between conditions on rankings of RSD per compound and the detected normalized area per compound. The RSDs were ranked in decreasing order (a low RSD signals good repeatability), while areas were ranked in increasing order (higher intensity equals greater sensitivity). Missing values were ignored. Statistical significance was set at 95 % (*p* ≤ 0.05). The Pearsons Chi/squared test was used to test statistical significance (*p* ≤ 0.05) of missing values in the on-line and off-line conditions. 

## Figures and Tables

**Table 1:** Combined results for the optimization of the on-line parameters for derivatization. The 1:10 calibration mix dilution was used.

Parameter-	MeOx Volume	Time	Temperature	Equilibration
Analyzed replicates	20 µL: 5 40 µL: 5 60 µL: 4	30/30 min: 4 60/30 min: 3 90/60 min: 5	30 °C: 4 37 °C: 3 45 °C: 3	0 h: 4 2 h: 4 4 h: 5 8 h: 4
Detected compounds	20 µL: 32 40 µL: 33 60 µL: 32	30/30 min: 33 60/30 min: 34 90/60 min: 31	30 °C: 38 37 °C: 37 45 °C: 36	0 h: 34 2 h: 36 4 h: 36 8 h: 34
Median RSD (%)	20 µL: 17 40 µL: 27 60 µL: 33	30/30 min: 23 60/30 min: 14 90/60 min: 18	30 °C: 10 37 °C: 10 45 °C: 21	0 h: 11 2 h: 21 4 h: 15 8 h: 15

**Table 2:** Repeatability and reproducibility test. Results are from plasma and liver (single batch) and pooled serum quality control (QC) samples (analyzed over three large batches of around 115 samples per batch). RSD: relative standard deviation.

**Parameter**	Plasma	Liver	Batch 1	Batch 2	Batch 3
Number of metabolites	22.8 ± 0.5	37 ± 0	20.3 ± 1.9	20.2 ± 2.4	20.2 ± 2.2
Number of missing values	11 (0.8%)	0 (0%)	24 (11%)	25 (12%)	25 (12%)
Median RSD	16%	10%	21%	20%	19%
RSD range	11–28%	2–56%	3–42%	12–69%	13–39%

**Table 3:** Comparison of on-line and off-line settings. RSD: relative standard deviation.

Parameter	On-Line	Off-Line (Original)	Off-Line with On-Line Settings (OLOLP)
Replicates	9	9	8
Number metabolites	23.4± 0.73	24.2± 0.83	24.4± 0.71
Number of missing values	14 (6%)	7 (3%)	5 (3%)
Median RSD	11%	21%	17%
RSD range	4–38%	4–48%	2–50%

## Data Availability

Data is contained within the article or supplementary material. Further data can provided upon request.

## Supplementary Materials

The following are available online at https://www.mdpi.com/article/10.3390/metabo11120888/s1, Supplementary Table S1. List of metabolite derivatives and their biological group used for reference search. MeOX: Methoxyamine hydrochloride. PPP: Pentose phosphate pathway. SCFA: short chain fatty acid. TCA: Tricarboxylic acid cycle. TMS: Trimethylsilyl derivatives. Supplementary Table S2. Composition of the calibration mix dilution for high (1:1), middle (1:10) and low (1:100) concentration (nM) used for each metabolite. Supplementary Table S3. Off-line and on-line parameters for optimization of the method. Supplementary Table S4. MeOx volume tested for optimization of derivatization conditions. Table of relative standard deviations (RSD) per compound and the median RSD overall and per biological classes in % for the scaled peak areas per metabolite. CMD: calibration mix dilution. Number of replicates vary due to occasional misinjections. Supplementary Table S5. Incubation time tested for optimization of derivatization conditions. Table of relative standard deviations (RSD) per compound and the median RSD overall and per biological classes in % for the scaled peak areas per metabolite. CMD: calibration mix dilution. Number of replicates vary due to occasional misinjections. Supplementary Table S6. Incubation temperature tested for optimization of derivatization conditions. Table of relative standard deviations (RSD) per compound and the median RSD overall and per biological classes in % for the scaled peak areas per metabolite. CMD: calibration mix dilution. Number of replicates vary due to occasional misinjections. Supplementary Table S7. Equilibration time tested for optimization of derivatization conditions. Table of relative standard deviations (RSD) per compound and the median RSD overall and per biological classes in % for the scaled peak areas per metabolite. CMD: calibration mix dilution. Number of replicates vary due to occasional misinjections. Supplementary Table S8. Individual relative standard deviation (RSD in %) per compound in *n* = 45 human plasma samples. AA: amino acids. TCA: Tricarboxylic acid cycle. Supplementary Table S9. Individual relative standard deviation (RSD in %) per compound in *n* = 18 mouse liver samples. AA: amino acids. TCA: Tricarboxylic acid cycle. Supplementary Table S10. Individual relative standard deviation (RSD in %) per compound in *n* = 9 pooled quality control samples from three batches. AA: amino acids. TCA: Tricarboxylic acid cycle. Supplementary Table S11. Ratios between the individual TMS groups for comparison of on-line, off-line and off-line with on-line conditions (OLOPL). Supplementary Figure S1. Concentration range of compounds in human plasma. A calibration mixture with high (1:1), middle (1:10) and low (1:100) concentrations were used (*n* = 5 replicates for calibration mixture and *n* = 45 for plasma). Supplementary Figure S2. Concentration range of compounds in human serum (pooled quality control samples). A calibration mixture with high (1:1), middle (1:10) and low (1:100) concentrations were used (*n* = 5 replicates for calibration mix and *n* = 9 serum samples). Supplementary Figure S3. Comparison of initial (starting on-line settings) and optimized (optimized on-line settings) auto sampler settings. The relative standard deviation of alkane 32 and the number of failed injections decreased from 68% to 8% and 18/59 (31%) to 0/42 (0%) in human plasma or solvent only, respectively. Supplementary Figure S4. Violin plots showing the distribution of the normalized peak area (log2 from metabolite normalized area ratio) compared to the original parameters. Supplementary Figure S5. Total ion chromatogram of a representative plasma sample analyzed with on-line derivatization. Insets display a composite of the extracted ion chromatograms for the top 3 masses for the requisite compounds. Compounds which have persistently poor peak shapes throughout a batch are normally removed by successive quality control checks before processing to statistical analysis.
