# Supplementary material for: Optimized Workflow for On-Line Derivatization for Targeted Metabolomics Approach by Gas Chromatography-Mass Spectrometry
Source: Metabolites. 2021 Dec 18;11(12):888. doi: 10.3390/metabo11120888 (PMC8703763; doi:10.3390/metabo11120888)
Supplement: Supplementary file 1 [file metabolites-11-00888-s001.zip › metabolites-1484219-supplementary.pdf]

metabolites

MDPI

Supplementary material for

Optimized Workflow for On-Line Derivatization for Targeted Metabolomics Approach by Gas Chromatography-Mass Spectrometry

Raphaela Fritsche-Guenther [1], Yoann Gloaguen [1,2], Anna Bauer [1], Tobias Opialla [1], Stefan Kempa [3], Christina A. Fleming [4], Paul H. Redmond [4], Jennifer A. Kirwan [1,*]

1 Berlin Institute of Health (BIH) @ Charité—Universitätsmedizin Berlin, BIH Metabolomics Platform, 13125 Berlin, Germany; Raphaela.fritsche@charite.de (R.F.-G.); yoann.gloaguen@mdc-berlin.de (Y.G.); anabauer@ual.com (A.B.); tobias.opialla@mdc-berlin.de (T.O.); jennifer.kirwan@charite.de (JA.K.)
2 Berlin Institute of Health (BIH) @ Charité—Universitätsmedizin Berlin, Core Unit Bioinformatics, 10117 Berlin, Germany
3 Max Delbrück Center for Molecular Medicine (MDC) in the Helmholtz Association, Berlin Institute of Medical Systems Biology, 10115 Berlin, Germany; Stefan.kempa@mdc-berlin.de
4 Department of Academic Surgery, Cork University Hospital, T12 DFK4 Cork, Ireland; christinafleming49@gmail.com (C.A.F.); henry.redmond@hse.ie (H.P.R.)
* Correspondence: jennifer.kirwan@charite.de

Supplementary Table S1. List of metabolite derivatives and their biological group used for reference search. MeOX: Methoxyamine hydrochloride. PPP: Pentose phosphate pathway. SCFA: short chain fatty acid. TCA: Tricarboxylic acid cycle. TMS: Trimethylsilyl derivatives.

Biological class	Metabolite	Detected as
Amino acid	Alanine	2TMS or 3TMS
Amino acid	Asparagine	2TMS
Amino acid	Aspartic acid	2TMS or 3TMS
Amino acid	Cysteine	3TMS
Amino acid	Glycine	2TMS or 3TMS
Amino acid	Isoleucine	1TMS or 2TMS
Amino acid	Leucine	1TMS or 2TMS
Amino acid	Lysine	3TMS
Amino acid	Methionine	1TMS or 2TMS
Amino acid	Phenylalanine	1TMS or 2TMS
Amino acid	Proline	1TMS or 2TMS
Amino acid	Serine	2TMS or 3TMS or 4TMS
Amino acid	Threonine	2TMS or 3TMS
Amino acid	Tryptophan	2TMS
Amino acid	Tyrosine	3TMS
Amino acid	Valine	1TMS, 2TMS
Glycolysis	Fructose-6-phosphate	1MeOx 6TMS
Glycolysis	Glucose-6-phosphate	1MeOx 6TMS
Glycolysis	Glyceric acid-3-phosphate	4TMS
Glycolysis	Lactic acid	2TMS
Glycolysis	Phosphoenolpyruvic acid	3TMS
Glycolysis	Pyruvic acid	1MeOx 1TMS
TCA	Citric acid	4TMS

TCA	Fumaric acid	2TMS
TCA	Glutaric acid, 2-hydroxy	3TMS
TCA	Glutaric acid, 2-oxo	1MeOx 2TMS
TCA	Malic acid	3TMS
TCA	Succinic acid	2TMS
Others (Nucleotide)	Adenine	2TMS
Others (Nucleotide)	Uracil	2TMS
Others (Nucleobase)	Adenosine	3TMS or 4TMS
Others (Nucleobase)	Cytosine	2TMS
Others (Glycerol)	Dihydroxyacetone phosphate	1MeOx 3TMS
Others (Glycerol)	Glycerol	3TMS
Others (Glycerol)	Glycerol-3-phosphate	4TMS
Others (Glycerol)	Glyceric acid	3TMS
Others (SCFA)	Butanoic acid, 3-hydroxy	2TMS
Others (SCFA)	Butanoic acid, 4-amino	3TMS
Others (Sugar alcohol)	Erythritol	4TMS
Others (Carboxylic acid)	Glutaric acid	2TMS
Others (PPP)	Ribose-5-phosphate	1MeOx 5TMS
Others (PPP)	Ribose	1MeOx 4TMS

Supplementary Table S2. Composition of the calibration mix dilution for high (1:1), middle (1:10) and low (1:100) concentration (nM) used for each metabolite.

Metabolite	1:1 (nM))	1:10 (nM)	1:100 (nM)
Adenine	7.40	0.74	0.074
Adenosine	18.71	1.87	0.187
Alanine	134.70	13.47	1.347
Asparagine	22.71	2.27	0.227
Aspartic acid	15.03	1.50	0.150
Butanoic acid, 3-hydroxy	28.82	2.88	0.288
Butanoic acid, 4-amino	9.70	0.97	0.097
Citric acid	52.05	5.21	0.521
Cysteine	8.25	0.83	0.083
Cytosine	9.00	0.90	0.090
Dihydroxyacetone phosphate	88.18	8.82	0.882
Erythritol	81.89	8.19	0.819
Fructose-6-phosphate	13.15	1.32	0.132
Fumaric acid	34.46	3.45	0.345
Glucose 6-phosphate	32.88	3.29	0.329
Glutaric acid	30.28	3.03	0.303
Glutaric acid, 2-hydroxy	57.27	5.73	0.573
Glutaric acid, 2-oxo	34.22	3.42	0.342
Glyceric acid	15.99	1.60	0.160
Glyceric acid-3-phosphate	43.48	4.35	0.435

Glycerol	65.15	6.52	0.652
Glycerol-3-phosphate	27.00	2.70	0.270
Glycine	66.60	6.66	0.666
Isoleucine	38.12	3.81	0.381
Lactic acid	446.19	44.62	4.462
Leucine	91.48	9.15	0.915
Lysine	20.53	2.05	0.205
Malic acid	44.75	4.47	0.447
Methionine	6.70	0.67	0.067
Phenylalanine	48.43	4.84	0.484
Phosphoenolpyruvic acid	15.04	1.50	0.150
Proline	60.80	6.08	0.608
Pyruvic acid	290.80	29.08	2.908
Ribose	19.98	2.00	0.200
Ribose-5-phosphate	91.21	9.12	0.912
Serine	114.19	11.42	1.142
Succinic acid	42.34	4.23	0.423
Threonine	167.90	16.79	1.679
Tryptophan	9.79	0.98	0.098
Tyrosine	11.04	1.10	0.110
Uracil	26.76	2.68	0.268
Valine	42.68	4.27	0.427

Supplementary Table S3. Off-line and on-line parameters for optimization of the method.

	Parameters	Off-line	On-line	On-line optimized	Remarks
MeOx/pyridine	Volume (µL)	20	20	20	Tested
	Time (minutes)	90	90	60	Tested
	Temperature (°C)	30	37	30	Tested
	Shaking	Thermoshaker 800 rpm	4 min quick mix 1,500 rpm followed by agitator 750 rpm	Agitator 250 rpm	Reduces spread of sample and white ring formation due to high speed of mixing.
	Preparation	Same time for all samples	Fresh every 24 hours	At the beginning preparation of all aliquots	After addition of fresh MeOx the peak areas of alkane 32 decreased.
MSTFA	Volume (µL)	80	80	80	
	Time (minutes)	60	60	30	Tested
	Temperature (°C)	37	37	30	Tested
	Shaking	Thermoshaker 800 rpm	4 min quick mix 1,500 rpm followed by agitator 750 rpm	Agitator 250 rpm	Reduces spread of sample and white ring formation due to high speed of mixing.
	Preparation	Same time for all samples	Fresh every 24 hours	At the beginning preparation of all aliquots	
Equilibration time (hours)		Variable for all samples	4	4	Tested
10 µL syringe		Wash after injection	Wash after injection	Wash after injection	
Fill speed		30 µL/s	30 µL/s	10 µL/s	Reduces misinjections
Eject speed		50 µL/s	50 µL/s	30 µL/s	Reduces misinjections
Fill stroke		5 µL	5µl	10 µl	Improved mixing of MeOx/Pyridine
Fill volume		1 µL	1 µL	2 µL	Reduces air bubbles and misinjections
100 µL syringe		Not used	No washing step	Wash after injection	Prolongs lifetime of syringe
Fill speed			30 µL/s	10 µL/s	Reduces misinjections
Eject speed			50 µL/s	20 µL/s	Reduces misinjections

Fill volume	80 µL	90 µL	Reduces air bubbles

Supplementary Table S4. MeOx volume tested for optimization of derivatization conditions. Table of relative standard deviations (RSD) per compound and the median RSD overall and per biological classes in % for the scaled peak areas per metabolite. CMD: calibration mix dilution. Number of replicates vary due to occasional misinjections.

**Metabolite/ biological class**	**Volume**	**20 µL**	**40 µL**	**60 µL**	**20 µL**	**40 µL**	**60 µL**	**20 µL**	**40 µL**	**60 µL**
	**CMD**	**1**	**1**	**1**	**10**	**10**	**10**	**100**	**100**	**100**
	**Replicates**	**5**	**5**	**5**	**5**	**5**	**4**	**5**	**5**	**4**
Asparagine	AA	47	74	27	14	47	46	NA	NA	NA
Lysine	AA	55	17	14	29	45	107	49	NA	7
Tryptophan	AA	56	47	29	NA	NA	NA	NA	NA	NA
Alanine	AA	18	26	30	47	33	30	66	84	34
Aspartic acid	AA	76	103	101	189	58	37	5	44	26
Glycine	AA	12	7	14	22	19	35	33	40	27
Isoleucine	AA	13	17	15	20	37	18	NA	NA	NA
Leucine	AA	11	3	13	12	20	27	48	51	12
Methionine	AA	21	11	14	NA	NA	NA	NA	45	7
Phenylalanine	AA	17	9	12	15	27	34	41	53	NA
Proline	AA	12	5	12	21	49	40	NA	NA	NA
Serine	AA	13	4	12	16	33	35	112	49	29
Threonine	AA	26	8	13	17	38	47	41	47	20
Valine	AA	10	4	12	11	18	22	35	37	47
Median RSD		17	10	14	18	35	35	41	47	26
Fructose-6-phosphate	Glycolysis	51	57	26	NA	94	NA	NA	NA	NA
Glucose-6-phosphate	Glycolysis	22	45	24	55	NA	NA	NA	NA	NA
Glyceric acid-3-phosphate	Glycolysis	54	51	26	54	42	66	NA	NA	NA
Lactic acid	Glycolysis	53	14	25	32	16	32	21	37	26
Phosphoenolpyruvic acid	Glycolysis	59	88	77	29	20	28	NA	NA	NA
Pyruvic acid	Glycolysis	56	67	40	16	42	50	84	53	20
Median RSD		54	54	26	32	42	41	52	45	23
Citric acid	TCA	54	27	10	18	35	47	26	15	19
Fumaric acid	TCA	10	5	10	11	13	17	31	11	32
Glutaric acid, 2-hydroxy	TCA	19	22	12	13	12	19	NA	NA	NA
Glutaric acid, 2-oxo	TCA	48	36	35	14	32	17	29	46	39
Malic acid	TCA	10	5	14	10	15	25	18	16	11
Succinic acid	TCA	9	3	12	12	14	24	11	13	14
Median RSD		14	13	12	12	15	22	26	15	19
Adenine	Others	49	52	NA	24	NA	NA	NA	NA	NA
Adenosine	Others	NA	27	NA	NA	NA	NA	NA	NA	NA
Cytosine	Others	26	44	36	NA	25	71	NA	NA	NA

Uracil	Others	9	7	11	12	26	38	55	25	52
Butanoic acid, 3-hydroxy	Others	8	2	40	11	7	20	17	17	28
Butanoic acid, 4-amino	Others	14	20	20	35	20	27	37	6	NA
Glycerol	Others	9	1	10	14	15	25	8	11	7
Glycerol-3-phosphate	Others	54	37	16	48	53	143	25	30	13
Glutaric acid	Others	11	3	12	10	13	19	13	15	16
Glyceric acid	Others	10	8	11	14	13	20	10	22	4
Ribose-5-phosphate	Others	93	15	49	31	66	112	NA	NA	NA
Ribose	Others	35	54	40	59	70	66	53	21	22
Median RSD		14	17	18	19	23	33	21	19	16
Median RSD all		21	17	15	17	27	33	32	34	20
Number compounds		37	38	36	33	33	32	24	24	22

Supplementary Table S5. Incubation time tested for optimization of derivatization conditions. Table of relative standard deviations (RSD) per compound and the median RSD overall and per biological classes in % for the scaled peak areas per metabolite. CMD: calibration mix dilution. Number of replicates vary due to occasional misinjections.

**Metabolite/ biological class**	**Time (min)**	**30/30**	**60/30**	**90/60**	**30/30**	**60/30**	**90/60**	**30/30**	**60/30**	**90/60**
	**CMD**	**1**	**1**	**1**	**10**	**10**	**10**	**100**	**100**	**100**
	**Replicates**	**5**	**3**	**5**	**4**	**3**	**5**	**4**	**3**	**3**
Asparagine	AA	82	46	12	12	12	54	NA	NA	NA
Cysteine	AA	NA	NA	2	NA	NA	NA	NA	NA	NA
Lysine	AA	13	19	10	60	NA	NA	19	69	NA
Tryptophan	AA	85	NA	85	44	76	58	NA	NA	NA
Tyrosine	AA	20	13	69	NA	43	NA	NA	NA	NA
Alanine	AA	7	12	6	20	25	19	23	74	20
Aspartic acid	AA	15	37	52	128	19	63	31	NA	NA
Glycine	AA	9	18	10	11	6	15	23	102	16
Isoleucine	AA	13	21	14	35	15	28	48	40	13
Leucine	AA	10	93	47	14	6	15	12	79	10
Methionine	AA	18	35	6	NA	28	NA	NA	NA	NA
Phenylalanine	AA	12	17	10	43	10	35	NA	NA	NA
Proline	AA	12	13	46	25	16	15	16	92	5
Serine	AA	8	13	11	13	6	11	17	93	19
Threonine	AA	10	19	13	19	14	19	31	83	16
Valine	AA	12	13	9	8	4	16	10	75	12
Median RSD		12	18	11	20	14	19	21	79	15
Fructose-6-phosphate	Glycolysis	16	22	11	NA	5	NA	NA	NA	NA
Glucose-6-phosphate	Glycolysis	26	26	5	NA	NA	NA	NA	NA	NA
Glyceric acid-3-phosphate	Glycolysis	18	5	56	37	45	23	NA	91	NA
Lactic acid	Glycolysis	43	15	21	84	46	62	83	10	12
Phosphoenolpyruvic acid	Glycolysis	16	31	20	8	7	29	NA	NA	NA

Pyruvic acid	Glycolysis	7	93	69	24	7	15	58	88	11
Median RSD		17	24	21	31	7	26	71	88	12
Citric acid	TCA	NA	NA	NA	NA	NA	14	NA	NA	NA
Fumaric acid	TCA	10	12	9	8	9	7	NA	56	8
Glutaric acid, 2-hydroxy	TCA	10	14	9	9	6	7	32	81	17
Glutaric acid, 2-oxo	TCA	11	81	56	26	79	8	NA	NA	NA
Malic acid	TCA	7	7	10	16	6	12	43	85	18
Succinic acid	TCA	33	37	58	144	95	70	68	77	25
Median RSD		13	17	12	23	14	18	27	76	16
Butanoic acid	Others	109	68	43	NA	NA	NA	NA	NA	NA
Butanoic acid	Others	25	48	16	23	130	45	22	27	46
Butanoic acid, 4-amino	Others	39	NA	NA	51	108	56	42	76	NA
Cytosine	Others	8	4	12	52	47	18	NA	NA	NA
Dihydroxyacetone-P	Others	8	12	NA	64	22	21	NA	NA	NA
Glutaric acid	Others	13	17	12	15	16	15	19	66	11
Glyceric acid	Others	42	13	10	12	10	12	10	48	NA
Glycerol	Others	9	13	8	6	5	14	31	55	27
Glycerol-3-phosphate	Others	67	NA	76	4	39	NA	37	66	NA
Ribose	Others	15	28	19	84	3	78	12	79	23
Ribose-5-phosphate	Others	31	10	7	104	NA	NA	4	83	NA
Uracil	Others	9	14	9	8	10	8	44	61	16
Median RSD		20	13	12	23	19	18	22	66	23
Median RSD all		13	18	12	24	15	17	23	77	16
Number compounds		37	34	36	33	34	31	24	25	19

Supplementary Table S6. Incubation temperature tested for optimization of derivatization conditions. Table of relative standard deviations (RSD) per compound and the median RSD overall and per biological classes in % for the scaled peak areas per metabolite. CMD: calibration mix dilution. Number of replicates vary due to occasional misinjections.

**Metabolite/ biological class**	**Tempera-tur**	**30 °C**	**37 °C**	**45 °C**	**30 °C**	**37 °C**	**45 °C**	**30 °C**	**37 °C**	**45 °C**
	**CMD**	**1**	**1**	**1**	**10**	**10**	**10**	**100**	**100**	**100**
	**Replicates**	**4**	**3**	**4**	**4**	**3**	**3**	**3**	**5**	**4**
Asparagine	AA	60	20	19	13	NA	103	NA	43	NA
Cysteine	AA	NA	29	28	NA	31	24	NA	NA	NA
Lysine	AA	64	9	17	21	12	17	35	73	59
Tryptophan	AA	NA	16	14	60	5	27	NA	67	59
Tyrosine	AA	NA	19	20	18	12	24	NA	76	47
Alanine	AA	19	11	18	48	41	20	45	NA	NA
Aspartic acid	AA	29	NA	NA	40	NA	NA	NA	NA	NA
Glycine	AA	15	NA	NA	7	NA	NA	23	NA	NA
Isoleucine	AA	30	7	21	39	1	13	8	62	NA
Leucine	AA	17	10	13	7	4	17	24	85	60

Methionine	AA	31	12	19	25	57	84	NA	NA	46
Phenylalanine	AA	20	10	14	8	11	27	62	70	64
Proline	AA	16	10	15	22	10	23	88	72	58
Serine	AA	15	10	12	8	5	13	72	67	44
Threonine	AA	68	7	12	10	6	19	47	73	65
Valine	AA	18	11	21	8	6	15	38	64	69
Median RSD		20	11	17	18	10	21	41	70	59
Fructose-6-phosphate	Glycolysis	30	19	20	11	10	14	35	NA	NA
Glucose-6-phosphate	Glycolysis	83	37	14	56	16	38	28	73	19
Glyceric acid-3-phosphate	Glycolysis	59	15	19	9	2	14	93	77	61
Lactic acid	Glycolysis	31	18	20	39	3	11	70	81	59
Phosphoenolpyruvic acid	Glycolysis	20	11	14	19	6	19	NA	79	64
Pyruvic acid	Glycolysis	25	49	48	21	10	NA	57	NA	NA
Median RSD		30	18	20	20	8	14	57	78	60
Citric acid	TCA	55	21	40	11	21	87	46	NA	NA
Fumaric acid	TCA	23	17	13	5	2	11	25	43	NA
Glutaric acid, 2-hydroxy	TCA	22	8	11	6	17	6	32	56	39
Glutaric acid, 2-oxo	TCA	40	16	13	13	2	24	143	77	65
Malic acid	TCA	14	19	12	7	3	24	64	70	63
Succinic acid	TCA	17	10	12	6	15	18	29	77	69
Median RSD		23	16	13	7	9	21	39	70	64
Butanoic acid, 3-hydroxy	Others	50	12	12	6	4	16	117	79	62
Butanoic acid, 4-amino	Others	22	30	16	4	16	10	36	66	58
Dihydroxyacetone-P	Others	NA	28	22	22	45	45	NA	77	69
Glycerol	Others	57	64	60	4	13	27	77	67	71
Glycerol-3-phosphate	Others	29	11	13	9	12	26	96	75	56
Adenine	Others	47	93	NA	NA	15	NA	NA	39	NA
Cytosine	Others	18	9	12	39	5	25	NA	66	60
Uracil	Others	20	10	NA	11	NA	NA	107	NA	NA
Glutaric acid	Others	44	20	49	5	35	19	101	61	60
Glyceric acid	Others	24	16	27	4	85	38	24	NA	NA
Ribose-5-phosphate	Others	37	14	11	9	6	26	64	68	58
Erythritol	Others	NA	16	19	NA	38	50	NA	8	47
Ribose	Others	54	16	11	9	6	22	53	67	31
Median RSD		37	16	16	9	14	26	77	67	59
Median RSD all		29	16	16	10	10	22	50	70	59
Number compounds		36	39	38	38	37	36	32	31	29

Supplementary Table S7. Equilibration time tested for optimization of derivatization conditions. Table of relative standard deviations (RSD) per compound and the median RSD overall and per biological classes in % for the scaled peak areas per metabolite. CMD: calibration mix dilution. Number of replicates vary due to occasional misinjections.

**Metabolite/ biological class**	**Time**	**0 h**	**2 h**	**4 h**	**8 h**	**0 h**	**2 h**	**4 h**	**8 h**	**0 h**	**2 h**	**4 h**	**8 h**
	**CMD**	**1**	**1**	**1**	**1**	**10**	**10**	**10**	**10**	**100**	**100**	**100**	**100**
	**Replicate**	**4**	**4**	**5**	**4**	**4**	**4**	**5**	**4**	**4**	**2**	**4**	**4**

Asparagine	AA	32	20	25	5	18	43	18	19	NA	NA	NA	NA
Cysteine	AA	NA	NA	38	NA	NA	NA	NA	NA	NA	NA	NA	NA
Lysine	AA	57	11	26	20	22	23	31	25	29	24	8	11
Tryptophan	AA	74	61	49	78	NA	48	25	44	NA	NA	NA	NA
Tyrosine	AA	NA	NA	41	NA	40	NA	12	NA	NA	NA	NA	NA
Adenosine	AA	50	30	90	62	NA	NA	NA	NA	NA	NA	NA	NA
Alanine	AA	26	38	34	29	56	76	31	45	81	38	28	34
Glycine	AA	28	19	29	4	10	19	12	11	6	11	21	12
Isoleucine	AA	25	21	117	19	36	52	32	79	NA	NA	120	NA
Leucine	AA	20	3	26	6	5	82	6	10	12	3	35	10
Methionine	AA	33	25	30	11	NA	26	32	27	NA	NA	NA	NA
Phenylalanine	AA	15	7	26	7	7	14	7	7	NA	NA	NA	NA
Proline	AA	20	4	27	7	15	6	35	27	41	12	50	20
Serine	AA	19	7	25	9	7	13	9	12	31	78	45	7
Valine	AA	23	7	26	6	12	15	15	13	29	12	39	27
Median RSD		26	19	29	9	15	25	18	22	29	12	37	12
Fructose-6-phosphate	Glycolysis	18	9	26	6	14	79	39	17	NA	NA	NA	13
Glucose-6-phosphate	Glycolysis	15	6	22	9	5	47	25	50	NA	NA	28	31
Glyceric acid-3-phosphate	Glycolysis	14	12	30	10	24	5	10	17	NA	NA	78	21
Lactic acid	Glycolysis	31	15	40	10	14	30	27	77	56	8	58	38
Phosphoenol-pyruvic acid	Glycolysis	16	23	37	7	25	11	14	21	NA	NA	NA	NA
Pyruvic acid	Glycolysis	33	12	27	15	64	36	5	53	68	21	36	16
Median RSD		17	12	29	9	19	33	19	35	62	8	47	21
Citric acid	TCA	19	15	24	12	8	27	16	10	30	60	39	17
Fumaric acid	TCA	21	9	27	66	8	13	7	11	12	33	19	10
Glutaric acid, 2-hydroxy	TCA	17	9	26	11	4	13	11	6	12	NA	33	9
Glutaric acid, 2-oxo	TCA	16	6	24	13	18	10	9	15	30	72	57	7
Malic acid	TCA	19	9	26	11	5	15	12	4	31	31	27	16
Succinic acid	TCA	22	8	28	10	4	19	9	7	18	16	19	10
Median RSD		19	9	26	12	6	14	10	9	24	33	30	10
Adenine	Others	18	17	21	18	NA	60	39	NA	NA	NA	NA	NA
Butanoic acid, 3-hydroxy	Others	24	10	28	12	5	16	8	6	9	3	27	13
Butanoic acid, 4-amino	Others	21	16	28	4	10	27	16	14	11	28	21	18
Cytosine	Others	18	6	26	6	68	47	35	32	NA	NA	NA	NA
Dihydroxyacetone phosphate	Others	NA	NA	NA	NA	71	64	NA	NA	NA	NA	NA	NA
Glutaric acid	Others	23	7	27	11	5	16	11	7	20	11	21	8
Glyceric acid	Others	19	64	28	66	6	10	8	10	17	20	22	18

Glycerol	Others	21	63	60	63	60	17	7	8	14	3	21	44
Glycerol-3-phos-phate	Others	56	16	32	19	26	13	25	24	142	89	69	38
Ribose	Others	24	11	27	16	4	75	17	15	27	20	72	35
Ribose-5-phos-phate	Others	88	74	71	34	8	101	15	22	60	41	46	12
Uracil	Others	20	8	28	5	10	15	17	14	29	30	36	22
Median RSD		21	16	28	16	10	22	16	14	20	20	27	18
Median RSD all		21	11	28	11	11	21	15	15	29	21	35	16
Number compounds		36	36	38	36	34	36	36	34	25	24	27	27

Supplementary Table S8. Individual relative standard deviation (RSD in %) per compound in n=45 human plasma samples. AA: amino acids. TCA: Tricarboxylic acid cycle.

Metabolite	Biological class	RSD
Alanine	AA	28
Glycine	AA	17
Leucine	AA	18
Lysine	AA	17
Methionine	AA	19
Ornithine	AA	12
Phenylalanine	AA	12
Proline	AA	21
Serine	AA	16
Threonine	AA	27
Tryptophan	AA	24
Tyrosine	AA	26
Valine	AA	11
Lactic acid	Glycolysis	13
Pyruvic acid	Glycolysis	12
Citric acid	TCA	15
Malic acid	TCA	13
Succinic acid	TCA	16
Butanoic acid, 3-hydroxy	Otherrs	12
Erythritol	Otherrs	15
Glyceric acid	Otherrs	19
Glycerol	Otherrs	15
Ribose-5-phosphate	Otherrs	20

Supplementary Table S9. Individual relative standard deviation (RSD in %) per compound in n=18 mouse liver samples. AA: amino acids. TCA: Tricarboxylic acid cycle.

Metabolite	Biological class	RSD
Alanine	AA	13
Aspartic acid	AA	5

Cysteine	AA	12
Glycine	AA	4
Lysine	AA	7
Methionine	AA	25
Phenylalanine	AA	2
Proline	AA	21
Serine	AA	17
Threonine	AA	13
Tryptophan	AA	29
Tyrosine	AA	47
Valine	AA	10
Fructose-6-phosphate	Glycolysis	5
Glucose-6-phosphate	Glycolysis	47
Glyceric acid-3-phosphate	Glycolysis	7
Lactic acid	Glycolysis	22
Phosphoenolpyruvic acid	Glycolysis	19
Pyruvic acid	Glycolysis	28
Citric acid	TCA	3
Fumaric acid	TCA	6
Glutaric acid, 2-hydroxy	TCA	3
Glutaric acid, 2-oxo	TCA	10
Malic acid	TCA	2
Succinic acid	TCA	2
Adenosine	Others	7
Butanoic acid, 3-hydroxy	Others	3
Butanoic acid, 4-amino	Others	56
Cytosine	Others	23
Glutaric acid	Others	3
Glyceric acid	Others	10
Glycerol-3-phosphate	Others	3
Glycerol	Others	29
Ribose-5-phosphate	Others	22
Ribulose-5-phosphate	Others	5
Ribose	Others	29
Uracil	Others	30

Supplementary Table S10. Individual relative standard deviation (RSD in %) per compound in n=9 pooled quality control samples from three batches. AA: amino acids. TCA: Tricarboxylic acid cycle.

Metabolite	Biological class	Batch 1	Batch 2	Batch 3
Alanine	AA	18	35	22
Aspartic acid	AA	25	19	26
Glycine	AA	22	15	13
Leucine	AA	11	19	22

Lysine	AA	18	24	17
Methionine	AA	3	20	14
Ornithine	AA	19	24	19
Phenylalanine	AA	25	30	34
Proline	AA	42	69	32
Serine	AA	21	15	26
Threonine	AA	29	20	28
Tryptophan	AA	39	27	27
Valine	AA	24	32	27
Glucose-6-phosphate	Glycolysis	21	20	22
Lactic acid	Glycolysis	13	12	19
Pyruvic acid	Glycolysis	25	21	14
Citric acid	TCA	19	22	39
Malic acid	TCA	23	18	38
Succinic acid	TCA	27	31	14
Butanoic acid, 3-hydroxy	Others	14	20	20
Glyceric acid	Others	18	27	19
Glycerol	Others	26	10	13
Ribose	Others	12	16	31

Supplementary Table S11. Ratios between the individual TMS groups for comparison of on-line, off-line and off-line with on-line conditions (OLOPL).

Metabolite	Derivatives	On-line	Off-line	OLOPL
Leucine	1TMS/2TMS	0.23	0.14	0.13
Phenylalanine	2TMS/1TMS	1.68	0.58	0.62
Threonine	2TMS/3TMS	8.21	0.42	0.52
Valine	1TMS/2TMS	0.21	0.12	0.11

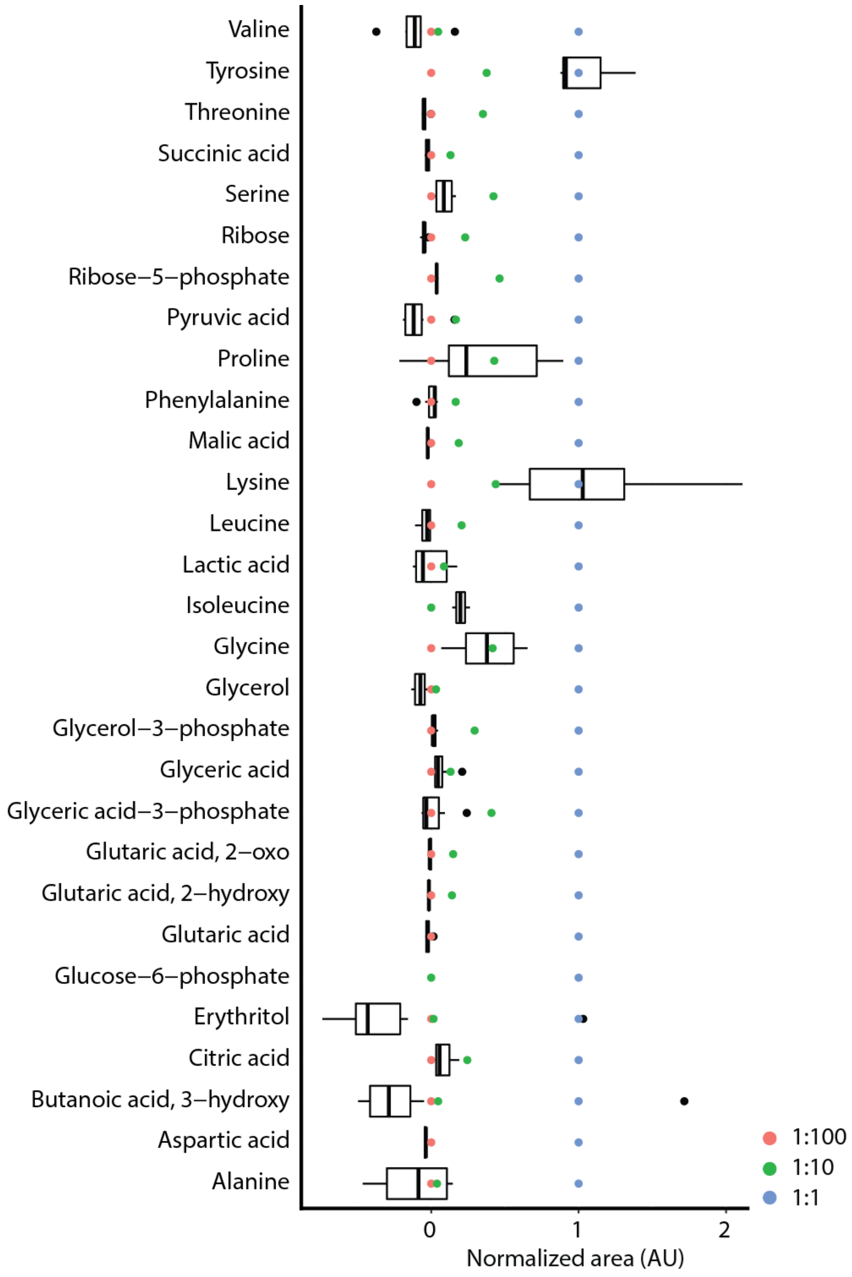

Supplementary Figure S1. Concentration range of 29/42 detected compounds in human plasma. A calibration mixture with high (1:1), middle (1:10) and low (1:100) concentrations were used (one replicates for calibration mixture and n=12 for plasma). A minimum/maximum normalization was performed on the calibration mixture for the single compounds.

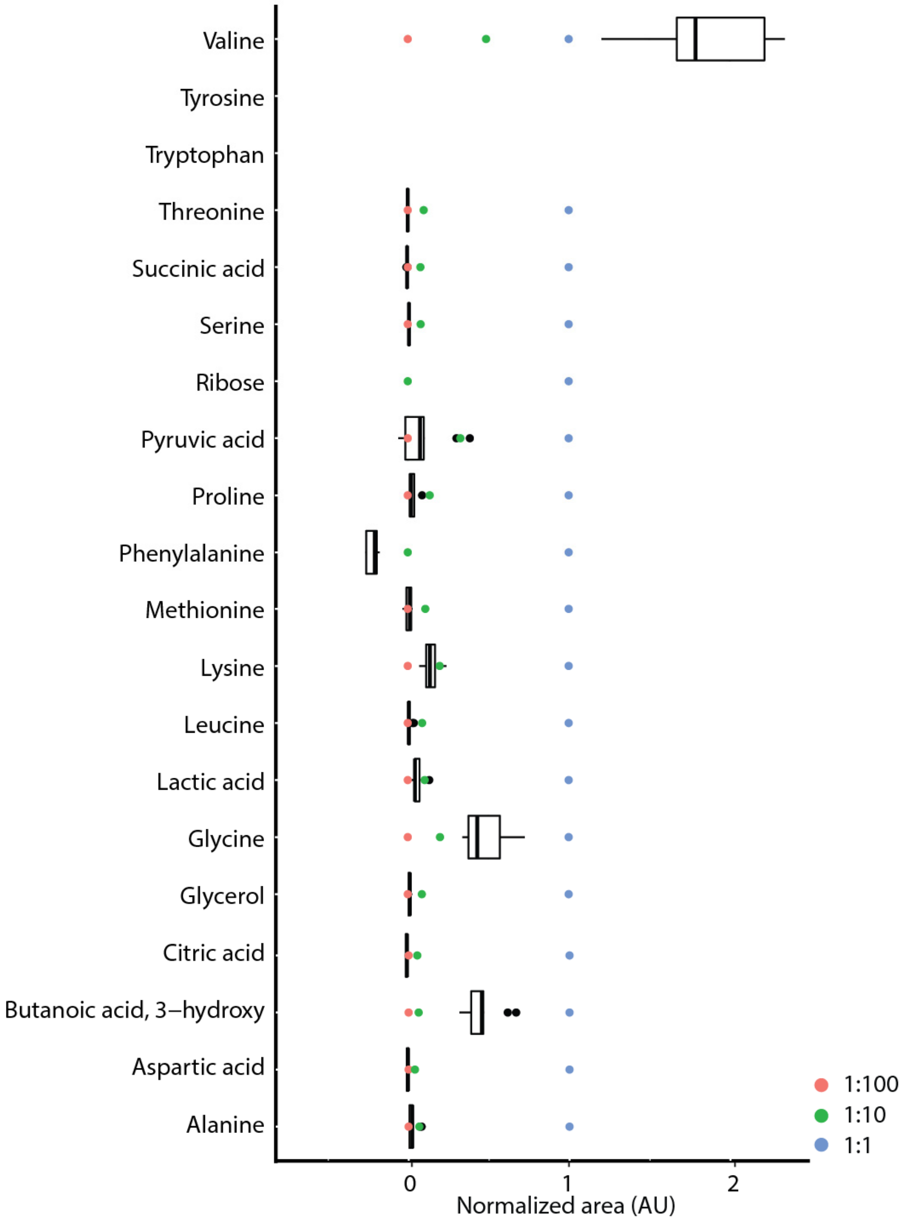

Supplementary Figure S2. Concentration range of 18/42 detected compounds in human serum (pooled quality control samples). A calibration mixture with high (1:1), middle (1:10) and low (1:100) concentrations were used (one replicate for calibration mix and n=9 serum samples). A minimum/maximum normalization was performed on the calibration mixture for the single compounds.

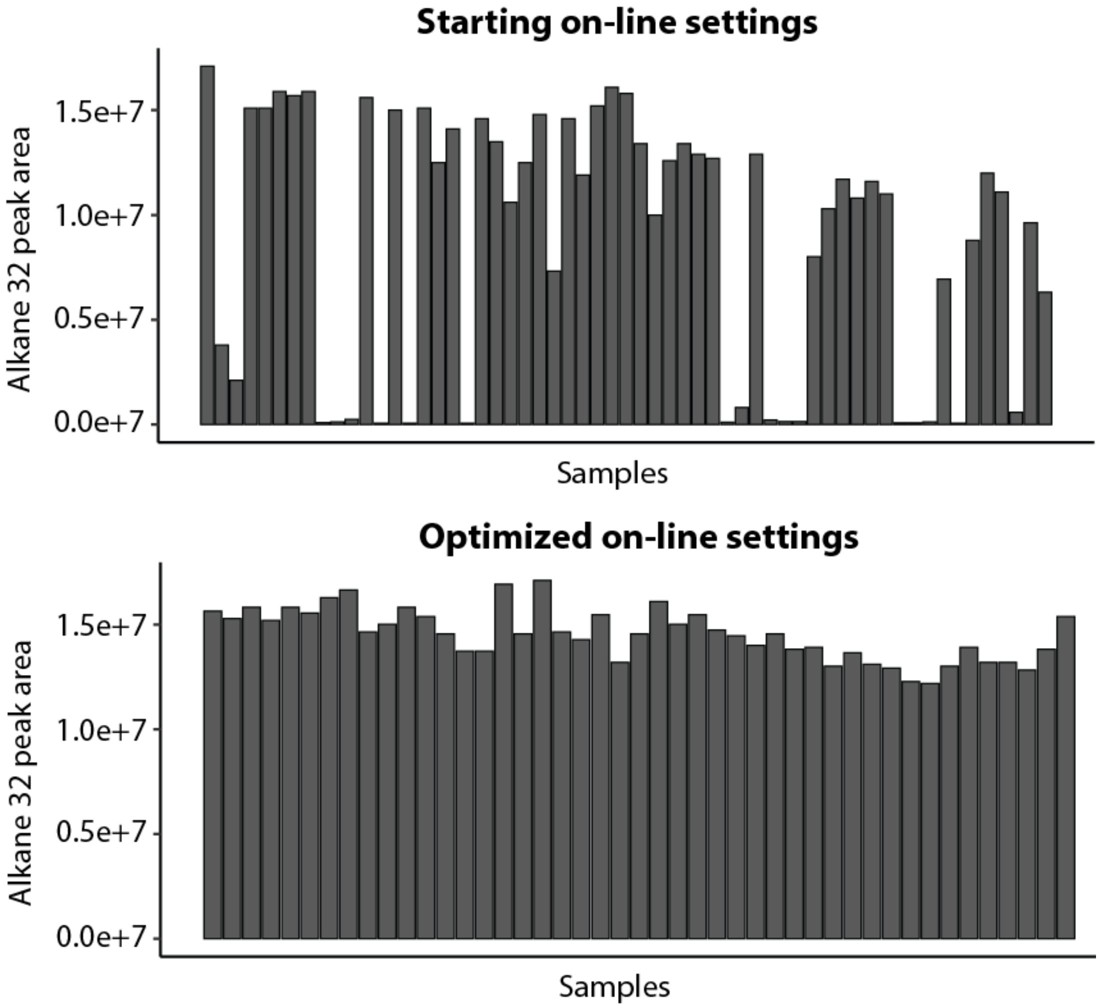

Supplementary Figure S3. Comparison of initial (starting on-line settings) and optimized (optimized on-line settings) auto sampler settings. The relative standard deviation of alkane 32 and the number of failed injections decreased from 68% to 8% and 18/59 (31%) to 0/42 (0%) in human plasma or solvent only, respectively.

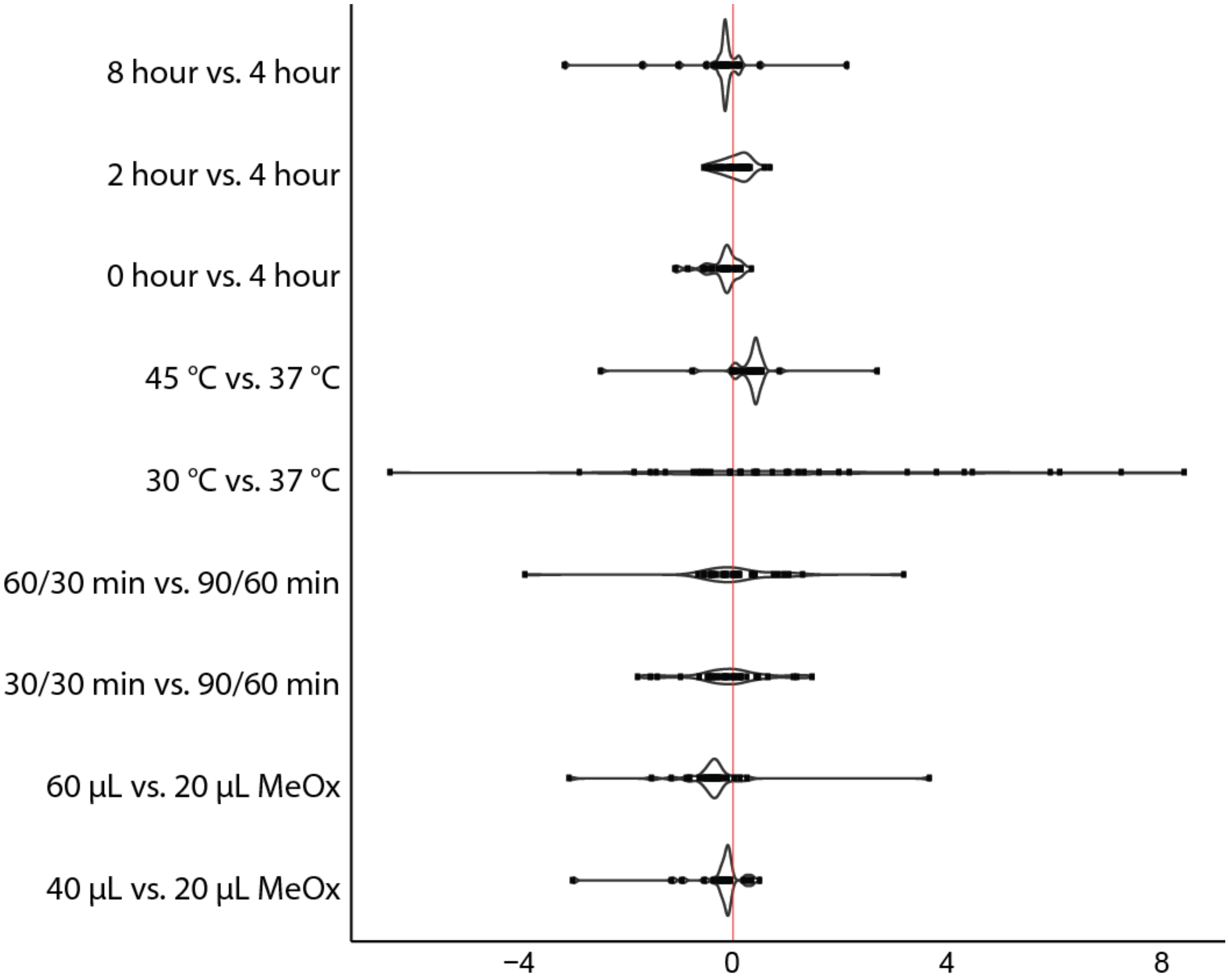

Supplementary Figure S4. Violin plots showing the distribution of the normalized peak area (log2 from metabolite normalized area ratio) compared to the original parameters.

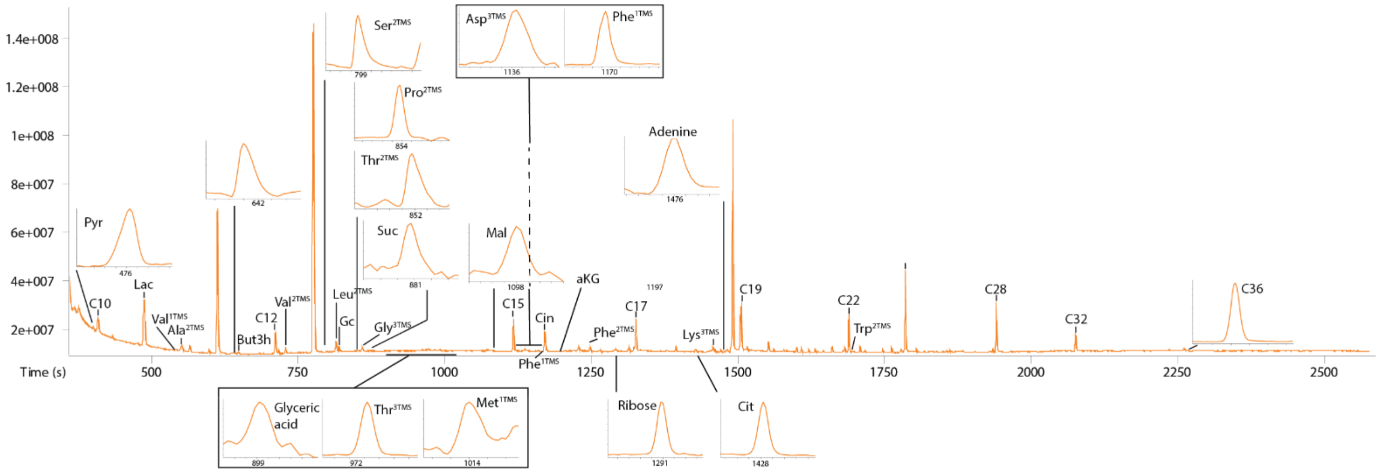

Supplementary Figure S5. Total ion chromatogram of a representative plasma sample analyzed with on-line derivatization. Insets display a composite of the extracted ion chromatograms for the top 3 masses for the requisite compounds. Compounds which have

persistently poor peak shapes throughout a batch are normally removed by successive quality control checks before processing to statistical analysis.
